# Diagnostic therapeutic assistance pathway (PDTA) of type 2 chronic rhinosinusitis

**DOI:** 10.3389/falgy.2023.1237131

**Published:** 2023-09-29

**Authors:** Frank Rikki Canevari, Alessia Giorli, Giulia Monti, Cesare Biagini, Diego Bagnasco, Carlo Cavaliere, Carlotta Pipolo, Eugenio De Corso, Matteo Gelardi, Giulia Gramellini, Alessandro Ioppi, Ignazio La Mantia, Luca Malvezzi, Maurizio Bignami, Puya Dehgani-Mobaraki, Veronica Seccia, Giandomenico Maggiore, Alberto Macchi

**Affiliations:** ^1^ Unit of Otorhinolaryngology – Head and Neck Surgery, IRCCS Ospedale Policlinico San Martino, Genoa, Italy; ^2^ Department of Surgical Sciences and Integrated Diagnostics (DISC), University of Genoa, Italy; ^3^ Otolaryngology Department, Azienda Ospedaliera Universitaria Senese, Siena, Italy; ^4^ ENT Department, ASST Sette Laghi, University of Insubria, Varese, Italy; ^5^ Allergy and Respiratory Diseases, Department of Internal Medicine (DIMI), IRCCS Policlinico San Martino, University of Genoa, Genoa, Italy; ^6^ Department of Sense Organs, Sapienza University of Rome, Rome, Italy; ^7^ Otolaryngology Unit, ASST Santi Paolo e Carlo, Department of Health Sciences, University of Milan, Milan, Italy; ^8^ Unit of Otorhinolaryngology and Head-Neck Surgery, Fondazione Policlinico A. Gemelli IRCCS, Rome, Italy; ^9^ Department of Otolaryngology, University Hospital of Foggia, Foggia, Italy; ^10^ ENT Deptartement, Asst Grande Ospedale Metropolitano Niguarda, Ear, Nose and Throat Unit of Sant'Orsola-Malpighi Hospital, Bologna, Italy; ^11^ Department of Otorhinolaryngology-Head and Neck Surgery, “S. Chiara” Hospital, Azienda Provinciale per i Servizi Sanitari (APSS), Trento, Italy; ^12^ Department of Medical and Surgical Sciences and Advanced Technologies “GF Ingrassia”, ENT Section, University of Catania, Catania, Italy; ^13^ Otorhinolaryngology, Head and Neck Surgery Unit, Humanitas Research Hospital, IRCCS, Milan, Italy; ^14^ Department of Otorhinolaryngology, Department of Surgery, ASST Lariana, University of Insubria, Como, Italy; ^15^ Ent and Head and Neck Surgery, Usl Umbria 1, Perugia, Italy; ^16^ Otolaryngology Audiology and Phoniatric Operative Unit, Department of Surgical, Medical, Molecular Pathology and Critical Care Medicine, Azienda Ospedaliero Universitaria Pisana, University of Pisa, Pisa, Italy; ^17^ Department of Otorhinolaryngology, Careggi University Hospital, Florence, Italy

**Keywords:** CRS, type 2 crs, biologic, pathway, multidisciplinary

## Abstract

Chronic rhinosinusitis (CRS) is a complex and heterogeneous disorder whose etiopathogenetic picture is not yet completely known and is classically divided into CRS with (CRSwNP) and without nasal polyps (CRSsNP). But today the distinction is made with type 2 and nontype 2 variants. A rational and defined pathway for the diagnosis of chronic rhinosinusitis is an indispensable means to be able to arrive at a correct identification of the patient. This typing is essential to be able to arrive at the correct course of treatment, which turns out to be different for different types of patients. For this reason, the realization of a diagnostic therapeutic pathway represents a fundamental way for the otolaryngologist specialist but not only, since today diagnostics has a multidisciplinary framework. In the present work, precise indications have been developed to arrive at a correct diagnosis. The various diagnostic pathways and processes to arrive at a correct therapeutic framing have been highlighted. Therapy ranging from medical therapy to surgical therapy without neglecting the new biological therapies. It does not represent a guideline but a diagnostic method that can be adapted to all the various territorial realities.

## 1. Introduction

Chronic rhinosinusitis (CRS) is a complex and heterogeneous disorder whose etiopathogenetic framework is not yet completely known and that is classically divided into CRS with (CRSwNP) and without nasal polyps (CRSsNP) (1).

Diagnostic Therapeutic Assistance Pathways (PDTA, in the Italian diction) represents a tool used all over the world to standardize at best the clinical approach to patients and specific conditions. Usually, PDTAs represent multidisciplinary instruments, as their development and application involve multiple specialists and caregivers. Actually, PDTAs can be produced as a regional/national tool, but many big care-providing structure prefer to have inner PDTAs, dedicated to specific medical problems. Ultimately, a PDTA has the ultimate aim to improve the provided and perceived quality of delivered care (2), and should allow to:

1. Estimate the total number of patients affected by a specific condition (prevalence);
2. Estimate the total number of new patients treated annually for a specific condition (incidence);
3. Define the temporal process of the services provided to patients, taking in consideration the level of care (prevention strategies, territorial healthcare, hospital care);
4. Define the outcomes of interest experienced by patients identifying appropriate and reliable outcome measures (indicators);
5. Estimate the resource consumption (in terms of tariffs but also in estimating real costs) of the PDTA;
6. Evaluate its practical effectiveness and efficiency;
7. Make comparisons to other PDTAs developed for the same health/care need, in terms of effectiveness, quality, and efficiency;
8. Carry out meta-analytical estimates and evaluations at national level.

Based on these premises, considering the significant prevalence of CRS in the Italian population and 53 the need for a structured, shared and joint diagnostic and therapeutic process, our group worked 54 to create a PDTA for CRS, and more specifically, for those characterized by a type 2 inflammation (3).

The purpose of this paper is to give guidelines for carrying out a proper therapeutic diagnostic course of type 2 rhinosinusitis. A pathway that can be adapted to the various local procedures in place while maintaining the proposed indications. Important, however, is the need not to see the problem of rhinosinusitis as solely the responsibility of the otolaryngologist but to consider that the patient should be analyzed from all points of view, involving other specialists; multidisciplinarity is essential to get to the goal.

## 2. The medical problem

The term rhinosinusitis identifies a state of acute or chronic inflammation of the mucosa the nasal cavities and paranasal sinuses (4).

This terminology arises from the consideration that both nasal and sinus mucosa are a single functional entity, overcoming the old concepts of “rhinitis” and “sinusitis”. Rhinosinusitis affects millions of people worldwide, both adults and children, and its incidence increased in recent years along with other forms of respiratory allergic disease. In fact, the disorder represents one of the main reasons for consulting the General Practitioner (GP) or the Pediatrician Specialist (5). Nevertheless, this trend may depend on many reasons, including the increased awareness of the disease and the new diagnostic criteria available (6).

CRS has an estimated prevalence that varies between 5% and 12% of the general population (10.9% European population, 13.4% American population). Instead, CRSwNP is thought to affect around 1.1% of the population in the USA, whereas estimates in Europe range between 2.1% and 4.4% (7). Such discrepancies depend on the definition of CRS that can rely on the presence of symptoms such as nasal obstruction, alteration of smell (and taste), rhinorrhea, facial pain, but also upon an objective endoscopic evaluation. Another element that may add confusion to the epidemiology of CRS is the use of paranasal sinuses Computerized Tomography (CT), where mild opacation of the sinuses can be considered indicative for CRS in otherwise asymptomatic patients (8).

However, confusion still exists about CRS management in the scientific community. As a result, this may translate for patients into numerous consultations (GP, Primary Pediatrician, Otolaryngologist, Allergologist, Pulmonologist) often without reaching a defined diagnosis in the correct time. Moreover, a clear and shared treatment plan is often missing.

From a surgical point of view, the management of these patients was profoundly changed in the last century by the discovery of mucociliary clearance, the concept of natural ostia and therefore by the introduction of functional endoscopic sinus surgery (FESS) (9). Despite the evolving technology and the spread of these mini-invasive techniques, recurrence of symptoms still represent an open issue. Indeed, a recent work observing a cohort of CRS patients over a 12-year period has revealed that 78.9% of patients with CRSwNP were subject to recurrence and 36.8% to revision ESS (10).

On the other hand, the new etiopathogenetic concepts of the so-called type 2 inflammation along with its therapeutic potential, has recently introduced another revolution in the way of seeing (and therefore managing) CRS. Indeed, the European Position Paper on CRS (EPOS) published in 2020 has introduced the new concept of endotype in CRS, so that both the condition and the patient are no longer classified only on the basis of the clinical features (phenotype) but also on the underlying type of inflammation. [EPOS2020] Generally, primary CRS (as opposed to CRS secondary to other conditions such as sinonasal tumors or cystic fibrosis, etc.) have been subdivided into type 2 dependent and non-type 2 forms (11). The type 2 inflammation is characterized by the prevalence of eosinophils (hence, the old name of eosinophilic CRS), the innate lymphoid cells type 2, and the interleukins 4, 5, and 13 (12). However, our knowledge remains limited as some patients with CRS may exhibit a type 1 (mostly neutrophilic) or type 3 inflammation (where IL-17 is preeminent), or even mixed endotypes (13).

This innovative classification of CRS has led to a consequent modulation of the therapies available to treat these specific form of CRS. Moreover, the patient can access primary, secondary (by means of a failure of medical therapy), but also tertiary (determined by the failure of surgical therapy) treatments. Subsequently, the clinicians and especially the rhinology experts must know how to adapt the available therapies in light of these criteria, and following a precise diagnostic-therapeutic algorithm.

## 3. PDTA: the diagnostic path

The creation of a sequential and structured path is fundamental to allow the correct endotyping of the pathogenetic mechanism and optimizing both the approaches to the disease and the cost of its management. For this purpose, diagnostic investigations can be divided into different levels of complexity and the shift to a higher level must be indicated by the results of the previous level. The same applies to treatment, where a step-up is decided according to the ineffectiveness of the therapeutic measures already adopted.

### 3.1. First level of investigation

These investigations allow the diagnosis of CRS and provide information on the severity of the disease. It must be kept in mind that many cases of CRS are self-treated by patients with over-the- counter medications such as saline irrigations or nasal decongestants (14).

From the results of first level investigations, a therapy or indication for second level investigations may be proposed. The first level consists of: confirmation of the diagnosis of CRS and collection of the past medical history, administration of patient-reported outcomes measures (PROMs) tests which investigate the impact of CRS on quality of life, and, finally, gather clinical objectivity such ash performing anterior rhinoscopy or nasal endoscopy.

#### 3.1.1. Medical history

When approaching a possible diagnosis of CRS the definition of rhinosinusitis provided by the EPOS 2020 guidelines must be rigorously applied. The guidelines define as CRS symptoms allowing to formulate a diagnosis the following: “presence of a nasal sinus inflammation characterized by the presence of two or more symptoms at least one of which must be nasal blockage / obstruction / congestion or rhinorrhea associated more or less with pressure or facial pain and more or less with reduction or loss of smell”. [epos2020] Other associated symptoms can be sore throat, cough, dysphonia, general malaise, or fever. Moreover, in order to define rhinosinusitis as chronic, symptoms must have been lasting for at least 12 weeks. [epos2020] The presence of atypical and/or localized symptoms should prompt the clinician towards other secondary forms (odontogenic sinusitis, sinonasal tumors, vasculitis etc.) (15, 16).

In order to better characterize the clinical phenotype and to identify possible factors affecting CRS control, it is essential to collect information on the presence of any comorbidities such as atopic dermatitis, asthma, urticaria, diabetes mellitus, and gastroesophageal reflux. The presence of one or more of these conditions can guide the diagnostic process (pinpointing towards type 2 inflammation) and must be considered when choosing the treatment strategy (e.g., long-term use of systemic steroids).

Information must also be collected on the patient’s general health conditions, paying particular attention to conditions that can lead to a diagnosis of secondary CRS as well as to comorbidities that can affect and/or limit the possible therapeutic options (e.g., uncontrolled diabetes mellitus, glaucoma, hypertension etc.).

The general drug history provides us with further information on the patient’s general state of health. The collection of anamnestic data on previous pharmacological treatments of rhinosinusitis must be conducted by differentiating between transnasal therapies (both in terms of duration and adherence to therapy) and systemic steroid therapies, by calculating the number of cycles per year and the dose taken/year. This last information is of fundamental importance as it allows to determine the effectiveness of the therapy as well as the excessive use of systemic steroids (17). Finally, data on previous surgical interventions must be careful collected by calculating the number, the type, the extent, as well as the time elapsed since the last surgical procedure performed (18).

#### 3.1.2. Quality of life questionnaires

Patients’ quality of life (QoL) should be the primary goal of every treatment; therefore, its quantification represents an essential step to manage correctly diseases such as CRS.

Numerous scales have been proposed for the quantification of symptoms, but no instrument or patient reported outcome measure (PROM) is completely satisfactory (19). The most used are the generic visual analog scale (VAS), the symptom-specific VAS and the Sino-Nasal Outcome Test-22 (SNOT-22).

- Generic VAS: on a scale from 0 to 10, where 0 corresponds to no impact, the patient is asked how much the symptoms of rhinosinusitis impact on their own quality of life.
- Symptom-specific VAS: on a scale from 0 to 10, where 0 corresponds to no impact, the patient is asked how much the single symptom of rhinosinusitis affects the quality of life.
- SNOT-22: it consists of 22 questions with categoric answers from 0 to 5, where 0 corresponds to no symptoms. The time period under consideration covers the last two weeks. Eight questions concern nasal symptoms, 4 refer to non-nasal symptoms and 10 on how much the symptoms impact on the psychophysical state (19).

#### 3.1.3. Clinical evaluation

Nasal endoscopy is a fundamental diagnostic step to accurately evaluate the nasal cavities and to define in detail the presence and type of any alteration (22). Additionally, the use of image enhancing filters such as Narrow Band Imaging (NBI) (Olympus Medical System Corporation, Tokyo, Japan) and Storz Professional Image Enhancement System (SPIES—Karl Storz, Tuttlingen, Germany), that emphasize the mucosal vascularization, can provide useful information for the differential diagnosis between inflammatory and neoplastic forms (23). Nasal endoscopy must be considered as a first level examination.

Endoscopic evaluation can be performed using rigid or flexible optics. When possible, rigid optical fibers are preferably used as they provide better image quality, allow for more precise examination of nasal cavities and meatuses and allow the use of the second hand for intranasal maneuvers such as aspiration of secretions and the collection of samplings for microbiological and / or histological examinations. The authors suggest a preparation of the nasal cavity with a mixture of decongestant and anesthetic in case of an examination with rigid optics, while the anesthetic can be avoided if using flexible scopes only: however, the evidence behind this practice is low at present (24).

The endoscopic evaluation is standardized in the so-called “three-pass technique”, which should be employed during all assessments. [epos2020] First, we must examine the respiratory district by evaluating the nasal floor, the inferior meatus, the inferior turbinate, the inferior portion of the nasal septum and the choana, starting from the nasal valve to the nasopharynx. The second pastime evaluates the medial compartment by visualizing the middle and upper portion of the nasal septum, the head of the middle turbinate, the olfactory cleft and the explorable portion of the ethmoid- sphenoid recess. The third step examines the lateral wall of the nose by evaluating the uncinate process, the explorable portion of the ostiomeatal complex, the lower portion of the middle turbinate, paying attention to its tail and the area of the fontanelles.

In case of CRS, the severity of the inflammatory process is examined with semi-quantitative staging systems.

**Figure 1:**
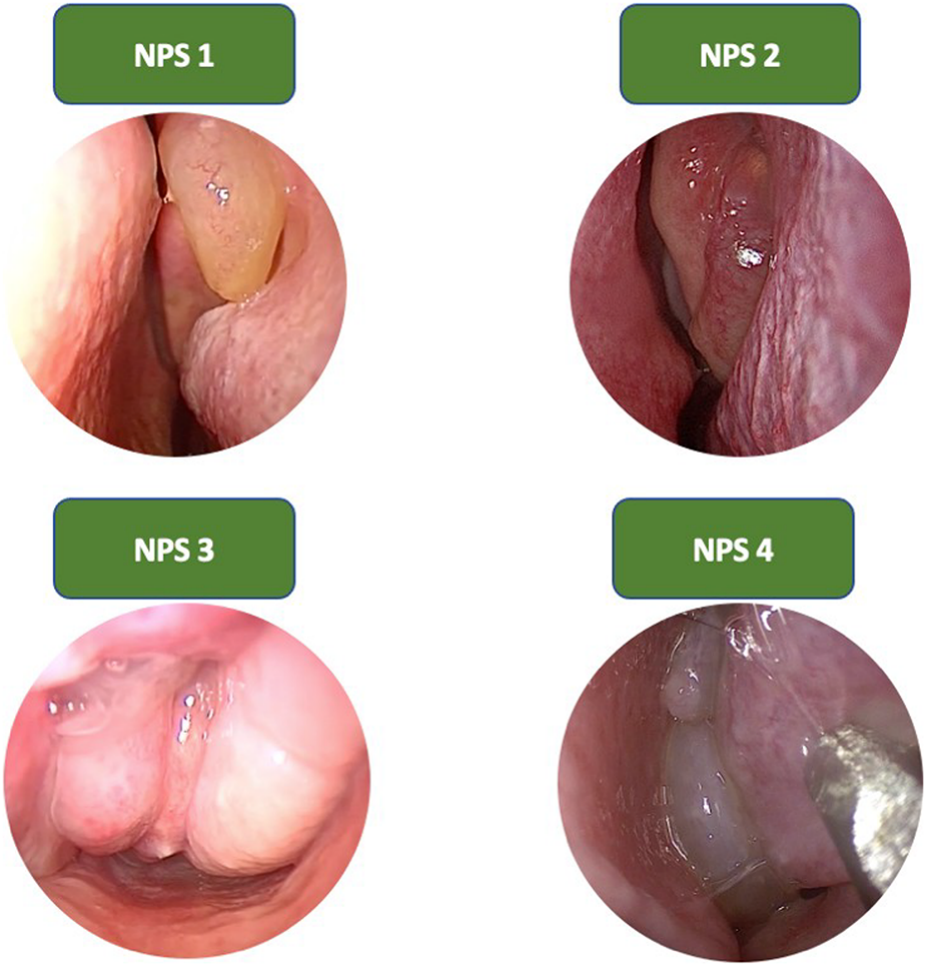
Nasal polipoid score.

The most used are:

• Nasal Polyp Score (NPS): it evaluates the extension of polypoid growths within the nasal cavities (25) (Figure 1). This system provides a grading from 0 to 4 for each nasal cavity, as follows:
  ◦ 0, polyp not visible;
  ◦ 1, small polyp confined within the middle meatus;
  ◦ 2, multiple polyps causing obstruction of the middle meatus;
  ◦ 3, polyps extending beyond the middle meatus, without complete obstruction or extending to the sphenoethmoid recess;
  ◦ 4, massive nasal polyposis.
• Lund Kennedy endoscopic score (26): it evaluates the severity of three objective data, for each nasal cavity:
  ◦ Nasal polyps:
     ▪ 0, (absence of polyps),
     ▪ 1, (polyps in the middle meatus),
     ▪ 2, (polyps extended to the nasal cavity);
   ◦ Edema:
     ▪ 0, (absent),
     ▪ 1, (mild/moderate),
     ▪ 2, (polypoid degeneration);
   ◦ Secretion:
     ▪ 0, (absent),
     ▪ 1, (serous),
     ▪ 2, (mucopurulent).

## 4. First level evaluation

First-level investigations tools allow to diagnose CRS and provide information on the severity of the disease. The first-level criteria that define rhinosinusitis as suitable for further investigations are those proposed by the European Forum for Research and Education in Allergy and Airway Diseases (EUFOREA) guidelines: SNOT-22 > 40, VAS > 5, NPS > 4, OCS > 1 course during the previous two years, at least one previous sinonasal surgery, presence of one or more comorbidities such as asthma, allergy.

### 4.1. Second level of investigation

Second level evaluations allow a typing (endotyping) of the immune inflammation that regulates the pathological process of CRS and allow to personalize the therapeutic choices.

In case of moderate to severe forms of CRS, both in non-endotyped patients and in already endotyped patients who have not improved in the first level assessments with the adequate therapy, further diagnostic tests are necessary.

The second level investigations include: nasal cytology, allergy tests, smell tests, radiological examinations and blood tests.

In addition, the severity and degree of control of asthma must be assessed though specific questionnaires, as it represents the main comorbidity of CRS.

#### 4.1.1. Nasal cytology

Nasal cytology allows the clinicians to understand the cellular composition of the nasal epithelium. Through this assessment, several conditions can be identified, such as: non-allergic rhinitis with eosinophils (NARES), non-allergic rhinitis with mast cells (NARMA), non-allergic rhinitis with neutrophils (NARNE) and eosinophil-mastcells non-allergic rhinitis—(NARESMA). Citology assessment is convenient, affordable, and provides several information that can help in the diagnosis and treatment of severe form of CRS. Precisely, the microscopical finding of eosinophils, mast cells, bacteria, spores and mycotic hyphae, is considered a sign of nasal pathology (27).

#### 4.1.2. Allergy tests

An accurate diagnosis of IgE-mediated sensitization to allergens is essential to quantify the prognosis and to guide the therapeutic choices in patients with CRS (28).

The main diagnostic method is represented by skin prick tests (SPT) and measurement of serum total and allergen-specific IgE. Basophil degranulation test (BAT) and nasal provocation tests remain third-level allergology investigations (29). As a working definition, the EPOS 2020 has proposed an IgE level of >100 UI as a possible marker for type 2 endotype. [epos2020].

SPT represents a cheap, easy to perform and to read, safe and quick evaluation. The allergen extracts used during the test must be relevant to the geographical area, thus the local pollen calendar should be consulted to set up a proper panel of allergens to test. The test is operator-dependent; thus, it must be performed by trained personnel. It is mandatory that a positive (histamine 0.1%) and a negative control are applied to rule out false positive and negative results (these latter may occur when the patient assumes antihistamines). It must be remembered that SPT use whole extracts, therefore they reveal the presence of IgE against all the components of the allergenic source, including cross-reactive proteins (30).

Finally, *in vitro* allergy diagnostics is indicated in cases of discrepancy between clinical history and SPT results, or when SPT cannot be performed (31).

#### 4.1.3. Olfactometric tests

Patients with CRS and allergies often live with debilitating olfactory disfunctions, indeed the prevalence of smell disorders is 60%–80% in these patients. Smell disorders can be divided into quantitative and qualitative disorders. Quantitative dysfunction includes: anosmia (complete loss of smell) and hyposmia (partial loss of smell). These can derive from various causes, the most common being viral infections, nasal sinus pathologies, hormonal disorders and head injuries, but they can also be a wake-up call for cognitive disorders such as Parkinson disease, Alzheimer disease or Multiple Sclerosis.

CRS-associated smell disorder has four peculiar clinical features: it is fluctuating, heavily steroid- dependent, it shows a pattern of low threshold and preserved identification scores, as well as it shows a preserved retro nasal olfactory function.

Clinical evaluation:

Several methods have been used to evaluate olfactory function or dysfunction:

1. Subjective evaluation: can be made by using proper questionnaires. Among these, the SNOT-22 is one of the most commonly used. It measures the loss of smell or taste (using a 5-point Likert scale) and the consequences of chronic rhinosinusitis such as reduced productivity, concentration and frustration.

Limitation: Not reliable, as self-assessment can underestimate the results in subjects with poor perception.

2. Objective evaluation: several chemosensory tests are performed to determine the precise nature, degree and veracity of the olfactory disorder, as well as favoring advice and monitoring the effectiveness of management strategies.

Psychophysical olfactory tests can be divided into threshold tests and above threshold categories. Threshold tests establish the lowest concentration of a smell that can be perceived (detection threshold) or recognized as quality (recognition threshold).

• Odor Detection Threshold Test: most popularly used due to the relatively high reliability and susceptibility to forced choice tests. In clinical threshold tests, phenylethyl alcohol (PEA) is the most commonly used odorant. Stimuli are manually presented using devices such as the sniffing test.
• Odor Discrimination Test: it evaluates whether, regardless of denomination or identification, a subject is able to perceive the differences between two or more odorants based on their quality.
• Odor identification test: patients are asked to identify the correct odor from the multiple choices provided.

#### 4.1.4. Radiological examinations

Radiological examinations are necessary to investigate the paranasal bony walls, and to get information that cannot be obtained with nasal endoscopy (such as the interface of polyps/neoformations with deep structures). Nose and paranasal sinuses CT scan is the gold standard of radiological examinations for nasal sinus pathologies. The methodology is now standardized as it involves image acquisition in the axial, coronal and sagittal projections with a slice thickness of <3 mm. Moreover, the bone and soft tissue window acquisitions are now commonly employed in every exam. This radiological assessment provides anatomical details that are fundamental for planning a surgical intervention. Maxillo-facial CT scan is also a useful tool for staging disease severity. Among the various radiological staging systems, the most used both in clinical trials and in daily clinical practice is the Lund Mackay score.

• Lund Mackay score: it is based on the evaluation of the opacification of the paranasal sinuses by assigning a score for each affected sinus ranging from: 0 no opacification, 1 partial opacification, 2 total opacification; with the exception of the ostiomeatal complex where 0 and 2 refers to clear and obstructed, respectively. The sum of the individual scores of both nasal cavities provides a value that is indicative of the severity of the disease. Chronic rhinosinusitis can be considered severe with a score > 12.
• The ACCES SCORE has been recently proposed as a staging system that allows to evaluate the completeness of previous surgeries, providing a predictive criterion for a future surgical revisions. This system evaluates the paranasal sinuses as follows: 0 no further surgery necessary, 1 surgery performed but not adequately, 2 no surgery performed. Again, the ostiomeatal complex represents an exception, since the only the values expected are 0 and 2.The comparison between Lund Mackay score and the ACESS score could be a useful tool to guide the most appropriate therapeutic strategy for the patient.

#### 4.1.5. Blood tests

The serum dosage of some parameters is of fundamental importance both in the diagnosis and endotyping of CRS and in the differential diagnosis of CRS with the vasculitic forms. However, the results of blood tests do not have an absolute value but must be correlated with the patient’s clinical history.

In primary CRS, total number of eosinophils >250 and total IgE >100 are considered pathological or suspected for a type 2 form. For lower values, however, a type 2 inflammation cannot be excluded as those could be related to a non-florid phase of the inflammation or could be caused by a recent systemic steroid therapy.

#### 4.1.6. Peak nasal inspiratory flow

The PNIF is a method that allows the measurement of the quantity of air that can pass through every single nostril. It measures nasal inhalation flow between 30 and 370 L/min. It uses a simple measurement of the speed at which air can move through the nose when inhaling forcefully.

#### 4.1.7. Asthma control test

The Asthma control test is a Tools that reflect the multidimensional nature of asthma control and that are easily and quickly administered and interpreted are needed to facilitate the assessment of asthma control in a busy clinical practice. The Asthma Control Test (ACT), a 5-item, patient-administered survey for assessing asthma control was developed to meet this need.

### 4.2. Third level of investigation

**Figure 2:**
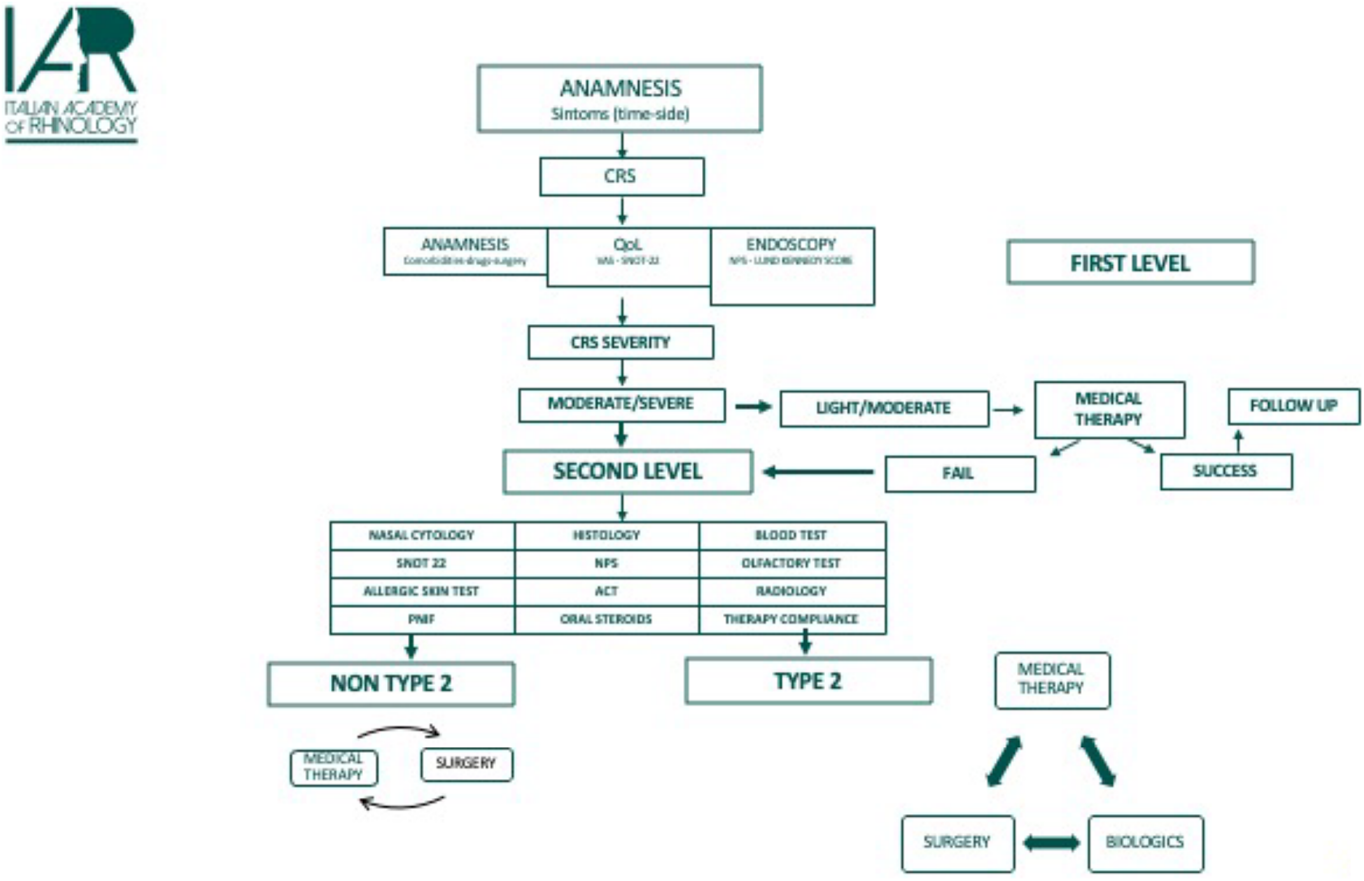
Diagnostic flowchart.

In response to a failure of the therapeutic and diagnostic path which followed the investigations of first and second level or in case of uncertain results, it is necessary to perform further multidisciplinary evaluation. Third-level investigations are directed towards the recognition of rare diseases which can mostly be classified as secondary CRS. Specialists who may be involved in this diagnostic step are pneumologists, allergologists, immunologists, pathologists, geneticists, nephrologists (Figure 2).

## 5. Treatment

The management of CRS patients must include a multidisciplinary approach. The treatment is established on the basis of the most recent EPOS and EUFOREA recommendations for the management of CRS; respecting the various endotypes, phenotypes and possible comorbidities present; recommending a progressive approach to pharmacological and surgical therapy. The setting up of a therapy by the caregivers, must take into account the concept of endotype of CRS, and therefore a precision medicine approach should be carried out.

To simplify and allow a wider application in clinical practice, the treatment paragraph of this PDTA will focus on the most widely used and supported by evidence therapies.

### 5.1. Local therapy

#### 5.1.1. Nasal corticosteroids

Different devices for the administration of local steroids (sprays, drops, aerosols, irrigations…), different molecules and different dosing schedules have been analyzed.

The results show that there are no significant differences with regard to the different molecules used, nor with regard to the efficacy, let alone the safety of the treatments.

Even the methods of drug delivery are equivalent, even if the nasal spray is certainly easier to administer and improves patient compliance. On the other hand, irrigations using a high-pressure nozzle may be useful in previously operated patients who have large anatomic spaces to reach with therapy.

Nasal corticosteroid administration improves quality of life and overall nasal symptoms when used over the long term and continuously. In patients with CRSwNP, it reduces polyp size and post- surgical recurrence (33).

#### 5.1.2. Nasal washes

Nasal washes or irrigations play an important role in the therapy of CRS. They have the role of removing crusts and mucus, improving muco-ciliary clearance, promoting ciliary beat activity, removing biofilm, allergens and inflammatory mediators present on the mucosa and improving hydration.

There are many devices on the market that deliver saline solution at different pressures. High- pressure nasal showers are more effective in the irrigation of maxillary sinuses and frontal recesses, especially in patients undergoing endoscopic surgery.

Hypertonic solution used as a spray has a better effect on nasal congestion and posterior nasal secretions with improvement of the associated cough symptom (34).

### 5.2. Systemic therapy

The most employed systemic treatments in CRS are systemic steroids, antibiotics and biologic treatments.

#### 5.2.1. Systemic corticosteroids

The rationale of systemic steroids relies in their high anti-inflammatory and anti-edemigenous activity, reducing the size of polyps and thus improving nasal patency.

No standard molecule is recognized as more effective than others, but prednisone is used in most clinical trials.

Even the dosage is not standardized and will depend on the patient’s comorbidities. On average, the period of administration is 14 days (in the literature it ranges from 7 to 21 days (35) using the highest dose for at least 5–6 days and therefore de-escalating the dosage.

It is important to remember that prolonged treatment with systemic corticosteroids should always be discouraged, as numerous side effects can occur, such as insomnia, mood swings, or elevations in blood pressure, but also more serious ones such as Cushing’s Syndrome, gastrointestinal disorders (even gastric ulceration), decompensated diabetes, favoring cataracts and osteoporosis. Finally, fatal herpes zoster cases have occurred (3). OCS are also recommended preoperatively before FESS because they reduce intraoperative bleeding and they reduce the operative time.

#### 5.2.2. Antibiotics

Their use is certainly central in the forms of flare-ups and the effectiveness is especially in cases without polyposis or however most of the work that have given a significance, uses the treatment without a phenotyping of CRS.

#### 5.2.3. Biologic drugs

The use of biologic drug therapy in CRSwNP from type 2 inflammation must follow international and national guidelines designed to administer the drug exclusively to patients who have a severe disease not responsive to standard drugs and/or who cannot benefit from surgical therapy. This particular attention is important to prevent overtreatment with drugs that, from current knowledge, are proposed as a treatment that is not “disease modifying” and must be taken long-term without the possibility of suspension. In addition, given the significant cost that is not yet comparable to standard therapy, even to a surgical procedure (36), the indication for treatment at the moment must be cautious.

At the moment, the available drug that can be prescribed for severe CRSwNP in Italy is Dupilumab 300 mg (biologic human antibody anti interleukin 4 and interleukin 13) via subcutaneous injection to be administered every 14 days. Other biological drugs, that have already been approved by the European Medicines Agency (EMA) are available, but not yet reimbursed by the Italian national health system, and therefore are not prescribed for the sole purpose of CRSwNP treatment. These are represented by anti-IgE (Omalizumab) and anti-interleukin 5 drugs (Mepolizumab-Benralizumab).

Based on the therapeutic plan established by the Italian Medicines Agency (AIFA), patients with the following characteristics are considered eligible for biologic treatment: age ≥ 18 years; endoscopic diagnosis of severe CRSwNP; NPS > 5 or SNOT-22 > 50; failure of prior medical treatments (at least 2 cycles of systemic corticosteroid in the last year); failure of previous surgical treatment (ascertained by the onset of post-operative complications or by lack of therapeutic response) (Table 1).

**Table 1:** Minimal criteria to prescribe biologics drug.

SNOT 22	>50
VAS	>7
NPS	>5
OCS	>2 course last year
Previous surgery	1 or more surgery
Comorbidities	Allergy asthma

The therapy response must be investigated after six months and at one year after starting the biologic treatment. Indeed, the physician should re-evaluate the patient and decide whether the response can be considered sufficient to warrant a long-term prescription.

From “ARIA-ITALIA Multidisciplinary consensus: nasal polyposis and biological drugs” (37).

### 5.3. Surgical therapy

Surgery for the treatment of CRS can be performed with different approaches, depending on the picture of clinical presentation and the prospect of recurrence of the single patient.

• Functional endoscopic nasal-sinus surgery (FESS).

This surgical approach is called “mucosal sparing” and is limited to clearing the nasal cavities and widening the ostia of the paranasal sinuses. This enables a correct ventilation of these latter and is indicated in patients with CRS from dysventilation of the sinuses, in patients with CRSwNP and possibly also in patients with CRSsNP with dominant neutrophilic inflammation. Furthermore, FESS allows local corticosteroids to penetrate deeper into the nasal cavities and in part to reach the sinuses and the sinus mucosa.

• Endoscopic Sinus Surgery (ESS) (20, 21).

This surgical approach is limited to deconstructing the nasal cavities and widening the sinus ostia in a way that is no longer “functional/mucosal sparing”. Furthermore, ESS is considered a disease modifier as it allows intranasal corticosteroids to penetrate into the sinuses itself and medication/washing to be performed in an outpatient setting (31).

• Reboot surgery

**Figure 3:**
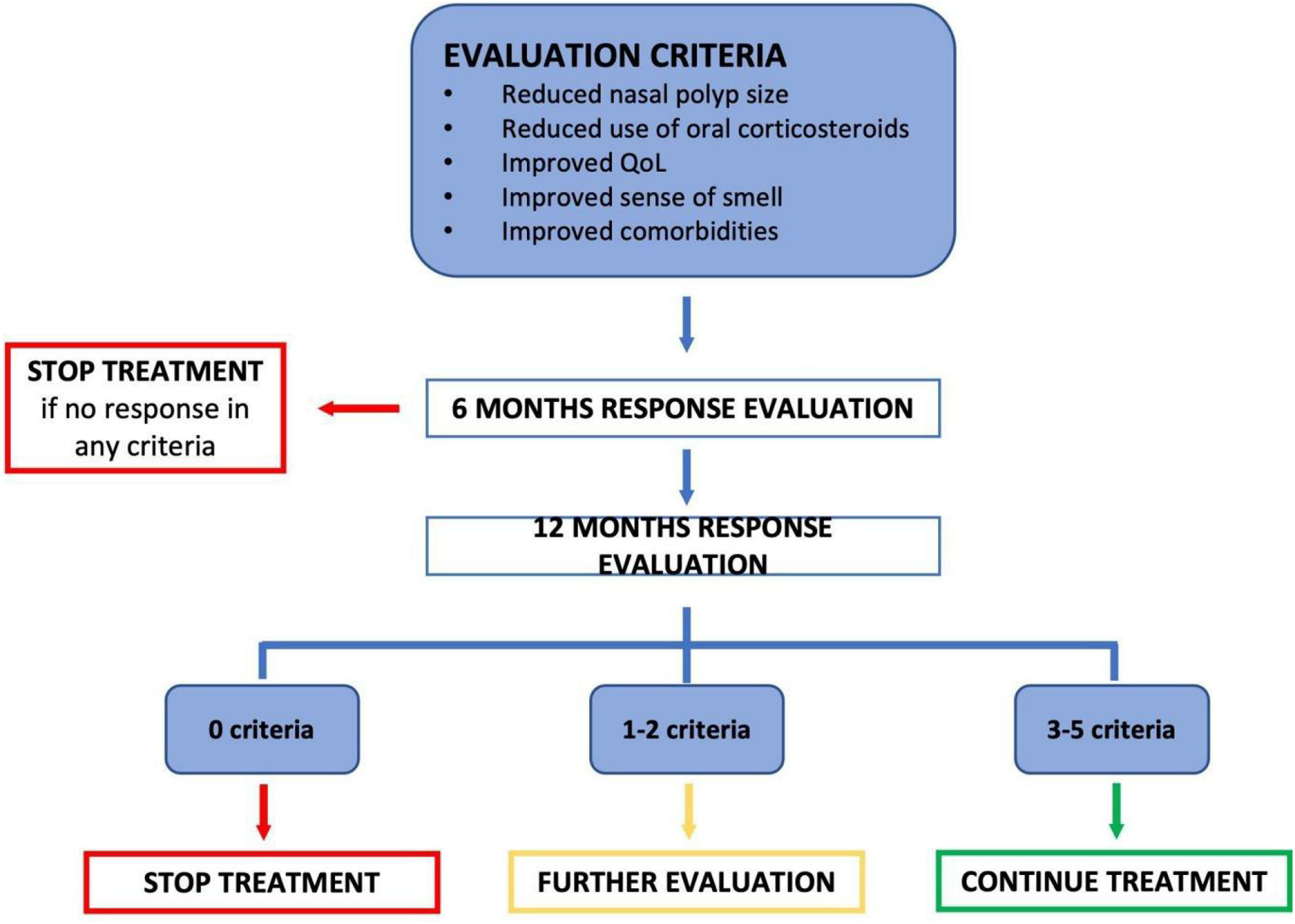
Evaluation Criteria.

This approach has recently been reintroduced by several authors (38), and focuses on the complete removal of all mucosa of the ethmoidal, frontal and maxillary sinuses, with the hypothesis that this in review approach should burden the regrowth of polypoid mucosa after the surgery. The indication for this type of surgery is seen for patients with severe CRSwNP and type 2 inflammation unresponsive to therapy approach should burden the regrowth of polypoid mucosa after the surgery (39) (Figure 3).

## 6. Conclusion

Implementing a therapeutic diagnostic pathway for chronic rhinosinusitis type 2 is an indispensable tool to arrive at the phenotyping and typing of the patient to set the correct treatment course. It is not a guideline but a pathway that can and should be adapted, keeping the basic points to one’s diagnostic needs. It represents a method validated and approved by a scientific society with its own medical-legal value. The pathway will be updated and modified whenever new scientific evidence on chronic rhinosinusitis type 2 intervenes. For this reason, the current working group proposes to intervene every three years to revise and update the pathway allowing a continuous revision phase.

## Data Availability

The original contributions presented in the study are included in the article/Supplementary Material, further inquiries can be directed to the corresponding author.
